# Triglyceride-glucose index is associated with severe obstructive coronary artery disease and atherosclerotic target lesion failure among young adults

**DOI:** 10.1186/s12933-023-02004-1

**Published:** 2023-10-21

**Authors:** Shalaimaiti Shali, Lingfeng Luo, Kang Yao, Xiangdong Sun, Hongyi Wu, Shuning Zhang, Lili Xu, Wei Gao, Jianxuan Li, Juying Qian, Yan Zheng, Yuxiang Dai, Junbo Ge, the GRAND investigators, Shalaimaiti Shali, Lingfeng Luo, Kang Yao, Xiangdong Sun, Hongyi Wu, Shuning Zhang, Lili Xu, Wei Gao, Jianxuan Li, Juying Qian, Yan Zheng, Yuxiang Dai, Junbo Ge

**Affiliations:** 1 Department of Cardiology, Zhongshan Hospital, Shanghai Institute of Cardiovascular Disease, Fudan University, Shanghai, 200032 China; 2 National Clinical Research Center for Interventional Medicine, Shanghai, 200032 China; 3 Human Phenome Institute, Fudan University, Shanghai, 200433 China; 4 Department of Cardiology, Xinyang Central Hospital, Xinyang Normal University, Xinyang, Henan 464000, China; 5 State Key Laboratory of Genetic Engineering, School of Life Sciences, Fudan University, Shanghai, 20438 China

**Keywords:** TyG index, Insulin resistance, Coronary artery disease, Early-onset, Target lesion failure

## Abstract

**Background:**

Early diagnosis and treatment effectiveness of early-onset coronary artery disease (EOCAD) are crucial, and non-invasive predictive biomarkers are needed for young adults. We aimed to evaluate the usefulness of the triglyceride-glucose (TyG) index, a novel marker of insulin resistance, in identifying young CAD patients and predicting their risk of developing target lesion failure (TLF).

**Methods:**

We recruited EOCAD patients (luminal narrowing ≥ 70%) and controls free from CAD (luminal narrowing < 30%), both aged 45 years or younger, from 38 hospitals in China between 2017 and 2020. EOCAD patients who underwent successful percutaneous coronary intervention were followed for incident TLF. TyG index was defined as Ln [fasting triglyceride (mg/dL) × fasting blood glucose (mg/dL)/2]. We used logistic regression and Cox proportional hazards modeling to evaluate the association of TyG index with prevalent EOCAD and incident TLF, respectively. The discriminatory ability of TyG index was assessed by the area under the receiver-operating characteristic curve (AUC).

**Results:**

Among the included 1513 EOCAD patients (39.6 ± 4.4 years, 95.4% male) and 1513 age-matched controls (39.0 ± 4.4 years, 46.4% male), TyG index was positively associated with the prevalence of EOCAD (adjusted odds ratio: 1.40, 95% confidence interval [CI] 1.23–1.60, per standard deviation [SD] increase in TyG index). The addition of TyG index to an empirical risk model provided an improvement in diagnostic ability for EOCAD, with a net reclassification improvement of 0.10 (95% CI 0.03–0.17, *p* = 0.005). During a medium of 33 month (IQR: 31–34 months) follow-up, 43 (3.3%) patients experienced TLF. Multivariate Cox regression model revealed that TyG index was an independent risk factor for TLF (adjusted hazard ratio [HR]: 2.410, 95% CI 1.07–5.42 comparing the top to bottom TyG index tertile groups; HR: 1.30, 95% CI 1.01–1.73, per SD increase in TyG index). Compared with a model of conventional risk factors alone, the addition of the TyG index modestly improved the AUC (0.722–0.734, *p* = 0.04) to predict TLF.

**Conclusions:**

TyG index is positively associated with prevalent EOCAD and incident TLF. TyG index appeared to be a valuable component of future efforts to improve CAD risk stratification and TLF outcome prediction among young adults.

**Supplementary Information:**

The online version contains supplementary material available at 10.1186/s12933-023-02004-1.

## Background

Atherosclerotic cardiovascular disease (ASCVD) is a leading cause of premature death worldwide [1]. The increase in early-onset coronary artery disease (EOCAD) has become an emerging public health concern [2–4]. Patients with EOCAD were more likely than their older counterparts to be treated with percutaneous coronary intervention (PCI) [3–7], target lesion failure (TLF), therefore, has been a focus of growing concern considering their life-long atherosclerotic burden [6, 7]. Early identifying young individuals at risk for CAD is of paramount importance for better prevention and management. With the recognition of the importance of modifiable risk factors, there have been renewing efforts toward a better understanding of cardiometabolic risk factors of CAD disease susceptibility and outcome [3–5]. Insulin resistance (IR) is the key precursor of a cluster of cardiometabolic abnormalities, including diabetes mellitus (DM), lipid disorders, arterial hypertension, and obesity, which in turn, play a joint role in the development of atherosclerotic CAD and may impact the outcome [8–10]. Triglyceride-glucose (TyG) index, as a novel marker of IR, has been proven to accurately and reliably reflect the degree of IR [11]. There has been accumulating evidence on the positive association of the TyG index with the prevalence of CAD and prognosis in the general population [12–15]. Nevertheless, little is known whether such an association exists among young individuals.

Herein, we conducted an analysis of the multicentre GRAND (clinical and genetic characteristics of coronary artery disease in Chinese young adults) study participants to examine whether the TyG index is associated with the prevalence of severe atherosclerotic obstructive CAD in young adults aged 45 years or younger and can also predict the occurrence of TLF after successful PCI.

## Methods

### Study population

The design and protocol of the GRAND study have been previously reported elsewhere [16]. Briefly, this is a nationwide, hospital-based clinical study. We recruited both EOCAD cases and age-matched controls at baseline, and then longitudinally followed the EOCAD patients only for their prognosis outcomes. From May 2017 to May 2020, a total of 2298 consecutive young patients aged 45 years or younger who underwent coronary angiography with or without PCI due to refractory myocardial ischemia or acute myocardial infarction (MI) were recruited from 38 hospitals in Chinese mainland. Only patients with severe atherosclerotic obstructive CAD (at least one major coronary artery with stenosis of ≥ 70% or ≥ 50% in the case of left main involvement) were included to the case group in this analysis. Young (≤ 45 years) controls free from CAD (luminal narrowing < 30%), confirmed by either invasive or computed tomography angiography, were also recruited from patients who were hospitalized at Zhongshan Hospital, Fudan University during the same period for other cardiac conditions (e.g., valvular heart disease, arrhythmia, cardiomyopathy and congenital heart disease, etc.). All participants were further excluded from this analysis if they had (1) suspected familial hypertriglyceridemia (triglyceride [TG] ≥ 5.65 mmol/L); or (2) body mass index (BMI) ≥ 45 kg/m2; or (3) fasting blood glucose (FBG) ≥ 22.2 mmol/L; or (4) myocardial ischemia or MI with non-obstructive CAD (stenosis < 50%) and spontaneous coronary artery dissection. Each case was randomly matched with a control in a 1:1 ratio based on age. Eventually, the baseline study population was consisted of 1513 EOCAD cases and 1513 young controls. Then, after excluding 211 patients who had severe obstructive EOCAD and did not undergo PCI at baseline, the remaining 1302 cases with successful PCI composited the post-PCI EOCAD cohort, and were followed up for 3 years. The study protocol was approved by the ethics committee at Zhongshan Hospital, Fudan University (B2017-051). All participants gave written informed consent before enrolment.

### Data collection

Data regarding the demographics and conventional CAD risk profiles were obtained for all eligible participants, whereas in-hospital information, including modes of clinical presentation, angiographic findings, medications, and choice of revascularization were collected for post-PCI patients by trained abstractors from electronic medical records. Additionally, the overall disease severity was quantified by the Genisini score as previously described [17]. Intervention strategies for target lesion, including percutaneous transcatheter angioplasty, drug-eluting balloon angioplasty, or drug-eluting stent implantation were at the physician`s discretion.

Blood samples were collected at 6 am after overnight fasting. The levels of biochemicals were measured routinely in the clinical laboratory of each hospital. The TyG index was calculated as Ln [fasting TG (mg/dL) × FBG (mg/dL)/2] [11]. Body mass index (BMI) was calculated as weight (kg) divided by the square of height (m^2^). BMI was classified into normal (BMI 18.5–23.9 kg/m^2^), overweight (BMI 24–27.9 kg/m^2^), and obesity (BMI ≥ 28 kg/m^2^) [18]. Estimated glomerular filtration rates (eGFR) were calculated with the Chronic Kidney Disease Epidemiology Collaboration equation, and chronic kidney disease (CKD) was defined as eGFR < 60 mL/min/1.73 m^2^. [19]. Hypertension was defined as a systolic blood pressure (SBP) ≥ 140 mmHg, a diastolic blood pressure (DBP) ≥ 90 mmHg, or a documented diagnosis of hypertension and/or treatment with hypertensive drugs. DM was defined as having an FBG ≥ 7.0 mmol/L, random plasma glucose ≥ 11.1 mmol/L, a hemoglobin A1c (HbA1c) ≥ 6.5%, or carrying the diagnosis of DM and/or treatment for DM. Dyslipidemia was defined as having a total cholesterol (TC) ≥ 5.2 mmol/L, or low-density lipoprotein cholesterol ≥ 3.4 mmol/L, or a TG ≥ 1.7 mmol/L, and/or a high-density lipoprotein cholesterol (HDL-c) < 1.29 mmol/L for women or < 1.03 mmol/L for men and/or on drug treatment.

### Follow-up

Post-PCI patients were followed for 3 years by clinical visits or re-hospitalization. Ascertainment of the prognosis outcomes was determined by a standardized physician review of all available medical records following the index admission. All patients underwent at least one repeated either invasive or computed tomography coronary angiography. Angiographic images were reviewed by two experienced interventional cardiologists blinded to patient`s clinical information. The outcome of interest was TLF which was defined as the combination of target vessel MI, or clinically driven target lesion revascularization due to > 50% angiographic restenosis of the target lesion, such as in-stent restenosis (> 50% stenosis within or immediately adjacent to a previously stented region), in-segment restenosis (> 50% stenosis anywhere between 5 mm from the proximal and distal edges of the stent), or stent thrombosis.

### Statistical analysis

The clinical characteristics of patients were presented for continuous variables as either the mean ± standard deviation (SD) or median with the interquartile range (IQR), depending on the normality of the data distribution, and the differences between groups were analyzed by either t-tests or Mann–Whitney non-parametric tests, and for categorical variables as absolute values (percentages) and the differences between groups were analyzed by Chi-square tests.

The association of EOCAD with standardized TyG index and tri-sectional TyG was evaluated using univariate and multivariate logistic models. Tests for linear trend were performed using the median value for each TyG tertile. The area under the receiver-operating characteristic curve (AUC) was used to determine the ability of different TyG index thresholds to discriminate between EOCAD cases and controls. Meanwhile, to evaluate whether the introduction of the TyG index into the model of established risk factors could improve the predictive value, the C-statistic was calculated and compared by DeLong’s test. In addition to AUC, net classification improvement (NRI) was calculated to evaluate whether TyG index materially affected reclassification performance, considering low sensitivity of AUC to detect the incremental predictive value of newly added indicator and its difficulty in clinical interpretation [20].

As a second step to evaluate the significance of the TyG index on TLF risk prediction, participants were categorized by the tertiles of the TyG index. Pearson's correlation analysis or Spearman's rank test was used, as appropriate, to determine the correlation between the TyG index and other baseline risk factors. The proportional hazards assumption of Cox models was tested based on the Schoenfeld residuals, and no evidence of violation was detected. Cox proportional hazards modeling for survival free from TLF was carried out by adjusting all baseline covariates that had a *p* < 0.05 in univariate association with the outcome, and other conventional risk factors. Stratified analyses were performed on participants according to their BMI, the presence of DM, modes of presentation, numbers of vessel lesions, Gensini score, intervention strategies of the target lesion, and the potential interaction effects between disease status and each above-mentioned variable were also examined. Moreover, the receiver-operating characteristic curve (ROC) analysis (discriminatory ability) and categorical NRI and IDI analysis (impact on risk prediction) was conducted as well. R 4.1.3 was used to carry out the above-mentioned analyses. A significance level of *p* < 0.05 (2-tailed tests) was applied.

## Results

### Baseline characteristics of study participants

The mean age of the study population was 39.3 ± 4.4 years, and 2146 (70.9%) of them were male. Baseline demographics, cardiovascular risk factors, and clinical characteristics were compared between young cases and controls in Table 1. Compared to young controls, EOCAD cases were more likely to be male, overweight, or obese, smokers and drinkers, and a patient with hypertension, DM, dyslipidemia, and CKD (*p* < 0.05). Restricting subjects to male participants (Additional file 1: Table S1) or to participants from Zhongshan Hospital, Fudan University (Additional file 1: Table S2) did not meaningfully change the differences in baseline characteristics between cases and controls.

**Table 1:** Baseline characteristics of study participants

Characteristics	Early-onset CAD N = 1513	Control N = 1513	*p-*value
General conditions			
Age, year	39.6 ± 4.4	39.0 ± 4.4	< 0.001
Male, n (%)	1444 (95.4)	842 (55.7)	< 0.001
BMI, kg/m^2^	26.4 ± 3.5	23.6 ± 3.7	< 0.001
Body shape, n (%)			< 0.001
Normal	342 (23.1)	878 (59.6)	
Overweight	685 (46.3)	386 (26.2)	
Obesity	451 (30.5)	210 (14.2)	
Heart rate	78.0 ± 13.2	78.9 ± 13.5	0.06
SBP, mmHg	128.0 ± 19.1	122.7 ± 14.7	< 0.001
DBP, mmHg	81.2 ± 13.9	78.1 ± 10.8	< 0.001
Smoke, n (%)			< 0.001
Never	382 (25.2)	1288 (85.1)	
Former	308 (20.4)	96 (6.3)	
Current	823 (54.4)	129 (8.5)	
Drink, n (%)	662 (43.8)	76 (5.0)	< 0.001
Comorbidities, n (%)			
Hypertension	669 (44.2)	377 (24.9)	< 0.001
DM	376 (24.9)	151 (10.0)	< 0.001
Dyslipidemia	873 (57.7)	592 (39.1)	< 0.001
Chronic kidney disease	39 (2.6)	21 (1.4)	0.03
Laboratory results			
TC, mmol/l	4.3 ± 1.6	4.2 ± 0.8	0.002
TG, mmol/l	2.3 ± 1.7	1.7 ± 1.3	< 0.001
HDL-C, mmol/l	1.0 ± 0.5	1.2 ± 0.4	< 0.001
LDL-C, mmol/l	2.7 ± 1.5	2.3 ± 0.7	< 0.001
Non-HDL-C, mmol/l	3.4 ± 1.6	3.0 ± 0.8	< 0.001
UA, mg/dl	6.5 ± 1.7	5.4 ± 1.7	< 0.001
Scre, mg/dl	0.9 ± 0.3	0.8 ± 0.3	< 0.001
eGFR, mL/min/1.73 m^2^	92.0 [82.1–103.6]	90.4 [82.6–97.9]	< 0.001
FBG, mg/dl	6.6 ± 2.8	5.3 ± 1.2	< 0.001
HbA1c, %	6.2 ± 1.7	5.4 ± 0.7	< 0.001
TyG index	9.2 ± 0.8	8.7 ± 0.7	< 0.001

Data were given as mean ± standard deviation, median with interquartile range or number (percentage)

*CAD* coronary artery disease, *BMI* body mass index, *SBP* systolic blood pressure, *DBP* diastolic blood pressure, *DM* Diabetes Mellitus, *TC* total cholesterol, *TG* triglyceride, *HDL-C* high-density lipoprotein-cholesterol, *LDL-C* low-density lipoprotein-cholesterol, *UA* uric acid, *Scre* serum creatinine, *eGFR* estimated glomerular filtration rate, *FBG* fasting blood glucose, *HbA1c* Glycosylated Hemoglobin Type A1C, *TyG* triglyceride-glucose

### Association of TyG index with EOCAD susceptibility

EOCAD cases presented a significantly higher level of the TyG index than young controls (mean ± SD, 9.2 ± 0.8 in cases vs. 8.7 ± 0.7 in controls, *p* < 0.001). After adjusting for age, sex, current smoking, drinking, hypertension, DM, and dyslipidemia, the TyG index was positively associated with the prevalence of EOCAD (odds ratios [OR]: 1.40, 95% confidence interval [CI] 1.23–1.60*, p* < 0.001, per SD increase in the TyG index). This corresponded to a twofold increased prevalence of EOCAD among participants in TyG index tertile 3 as compared to tertile 1 (OR: 2.00, 95% CI 1.48–2.71, *p* < 0.001) (Table 2). Among EOCAD patients, TyG index was significantly and positively correlated with BMI, SBP, DBP, TC, non-high-density lipoprotein-cholesterol (non-HDL-C), and HbA1c, but negatively correlated with HDL-C (all *p* < 0.001, Additional file 1: Table S3). The independent association observed between the TyG index and the likelihood of EOCAD did not alter when restricting participants to males (Additional file 1: Table S4).

**Table 2:** The association between the TyG index and prevalent EOCAD

TyG index	OR (95% CI)					
	Model1	*p*-value	Model2	*p*-value	Model3	*p*-value
Per SD increase	2.07 (1.91–2.26)	< 0.001	1.41 (1.27–1.56)	< 0.001	1.40 (1.23–1.60)	< 0.001
Tri-sectional TyG groups						
Tertile 1	1 (Reference)		1 (Reference)		1 (Reference)	
Tertile 2	2.11 (1.76–2.53)	< 0.001	1.13 (0.89–1.43)	0.31	1.18 (0.92–1.52)	0.19
Tertile 3	4.90 (4.06–5.92)	< 0.001	1.97 (1.55–2.52)	< 0.001	2.00 (1.48–2.71)	< 0.001
*p* for trend	< 0.001		0.003		0.01	

Model 1: unadjusted for covariates

Model 2: adjusted for age, sex, body mass index, current smoking, drinking

Model 3: adjusted for age, sex, body mass index, current smoking, drinking, hypertension, diabetes mellitus, and dyslipidemia

*TyG* triglyceride-glucose, *EOCAD* early-onset coronary artery disease, *OR* odds ratio, *CI* confidential intervals

### Diagnostic performance of TyG index for EOCAD susceptibility

**Fig. 1:**
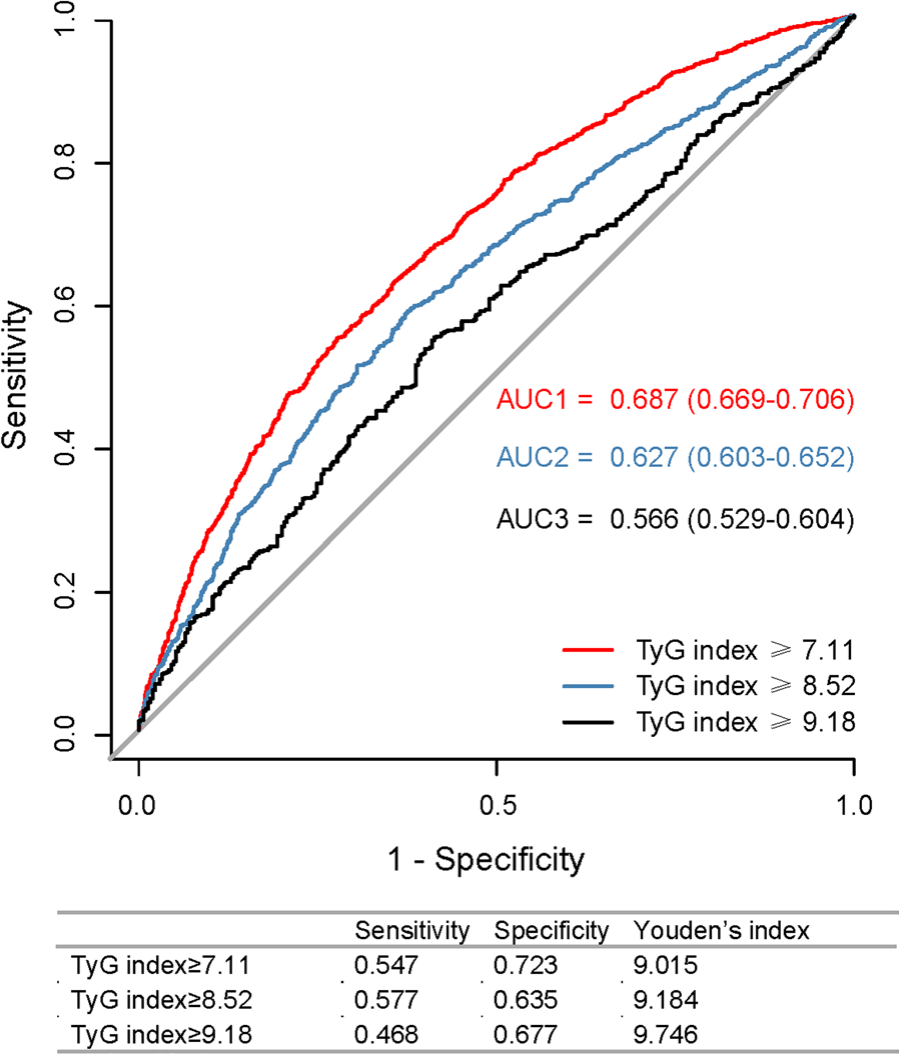
ROC analysis of TyG index at hospitalization by various rule-in thresholds to identify EOCAD among young adults. *ROC* receiver operating characteristic curve, *TyG* index triglyceride-glucose index, *EOCAD* early-onset coronary artery disease, *AUC* area under the curve

The ROC for the occurrence of EOCAD had the largest AUC of 0.687 (95% CI 0.669–0.706, *p* < 0.001) when the rule-in threshold of TyG index was 7.11, and had an AUC of 0.627 (95% CI 0.603–0.652, *p* < 0.001) and 0.566 (95% CI 0.529–0.604, *p* < 0.001) when the rule-in threshold of TyG index was 8.52 and 9.18, respectively (Fig. 1). The critical value of the TyG index to estimate EOCAD risk was 9.015 (sensitivity: 54.7%; specificity: 72.3%). Although there was no increment in AUC (from 0.897 to 0.889, *p* = 0.77), the addition of the TyG index to the established risk estimation model that included age, BMI, sex, current smoking, drinking, hypertension, DM, high levels of uric acid and non-HDL-c, and decreased levels of HDL-c, provided an improvement of diagnostic ability for EOCAD, with a continuous NRI of 0.1018 (95% CI 0.0307–0.1728, *p* = 0.005). This suggested an improved predictive power by the addition of the TyG index, which correctly reclassified EOCAD cases by 10.18%. IDI analysis did not show statistically significant improvement in reclassification (IDI: 0.0001, 95% CI − 0.0004–0.0013, *p* = 0.27). The sensitivity analysis in men showed the largest AUC of 0.621 (95% CI 0.594–0.674; *p* < 0.001) when the rule-in threshold of TyG index was 0.733, and the critical value was 9.183 (sensitivity: 50.0%; specificity: 70.3%) (Additional file 1: Fig S1).

### TyG-index and incident TLFs

Baseline characteristics of 1302 post-PCI patients were listed by tertiles of TyG index (Table 3). The most prominent differences were that patients in the highest tertile group were more likely to be male, obese, smokers or alcohol drinkers, tended to have hypertension, DM, and lipid disorders, showed higher heart rate, higher SBP, and DBP, and had worse lipid and glucose profile as compared to those in the lower tertile groups of TyG index. There were no significant differences across tertile groups of TyG index in the clinical presentation, left main/left anterior descending artery involvement, Gensini score, left ventricular ejection fraction (LVEF), and the course of PCI. Those in the highest tertile group of TyG index were more likely to have multivessel disease. The secondary prophylactic medications differed, with the lowest prescription rates occurring in the lowest TyG index tertile group. Additionally, the TyG index had no significant correlation with the Gensini score or LVEF (Additional file 1: Table S3).

**Table 3:** Baseline characteristics of EOCAD patients who underwent PCI stratified by tertiles of TyG index

	Overall N = 1302	Tertile 1 N = 435	Tertile 2 N = 433	Tertile 3 N = 434	*p*-value
General conditions					
Age, year	39.5 ± 4.5	39.7 ± 4.5	39.3 ± 4.5	39.5 ± 4.4	0.43
Female, n (%)	54 (4.1)	32 (7.4)	14 (3.2)	8 (1.8)	< 0.001
BMI, kg/m^2^	26.5 ± 3.5	25.6 ± 3.4	26.8 ± 3.4	27.2 ± 3.6	< 0.001
Body type, n (%)					< 0.001
Normal	285 (22.4)	137 (32.5)	76 (18.0)	72 (16.9)	
Overweight	598 (47.0)	194 (46.1)	212 (50.1)	192 (45.0)	
Obesity	388 (30.5)	90 (21.4)	135 (31.9)	163 (38.2)	
Heart rate	77.9 ± 13.0	76.2 ± 12.9	78.2 ± 12.9	79.3 ± 13.1	0.001
SBP, mmHg	128.0 ± 18.9	126.0 ± 17.7	127.2 ± 18.6	130.9 ± 19.9	< 0.001
DBP, mmHg	81.2 ± 13.8	80.0 ± 13.0	80.3 ± 13.3	83.3 ± 14.8	< 0.001
Smoking, n (%)					< 0.001
Never	321 (24.7)	142 (32.6)	93 (21.5)	86 (19.8)	
Former	270 (20.7)	91 (20.9)	94 (21.7)	85 (19.6)	
Current	711 (54.6)	202 (46.4)	246 (56.8)	263 (60.6)	
Drinking, n (%)	564 (43.3)	149 (34.3)	215 (49.7)	200 (46.1)	< 0.001
Comorbidities, n (%)					
Hypertension	566 (43.5)	176 (40.5)	176 (40.6)	214 (49.3)	0.01
DM	325 (25.0)	66 (15.2)	78 (18.0)	181 (41.7)	< 0.001
Dyslipidemia	763 (58.6)	121 (27.8)	302 (69.7)	340 (78.3)	< 0.001
Chronic kidney disease	31 (2.4)	10 (2.3)	6 (1.4)	15 (3.5)	0.13
History of previous MI	243 (18.7)	88 (20.2)	76 (17.6)	79 (18.2)	0.57
Laboratory results					
TC, mmol/l	4.4 ± 1.5	3.9 ± 1.4	4.5 ± 1.5	4.7 ± 1.6	< 0.001
TG, mmol/l	2.4 ± 1.7	1.1 ± 0.3	2.1 ± 0.6	3.9 ± 1.9	< 0.001
HDL-C, mmol/l	1.0 ± 0.5	1.2 ± 0.6	0.9 ± 0.3	0.9 ± 0.4	< 0.001
LDL-C, mmol/l	2.7 ± 1.5	2.6 ± 1.7	2.7 ± 1.3	2.6 ± 1.4	0.36
Non-HDL-C, mmol/l	3.4 ± 1.5	2.8 ± 1.3	3.6 ± 1.4	3.9 ± 1.5	< 0.001
UA, mg/dl	6.5 ± 1.7	6.4 ± 1.6	6.6 ± 1.6	6.4 ± 1.7	0.10
Scre, mg/dl	0.9 ± 0.2	0.9 ± 0.2	0.9 ± 0.2	0.9 ± 0.3	0.69
eGFR, mL/min/1.73 m^2^	92.3 [82.9–103.7]	92.3 [82.9–102.0]	91.8 [82.1–103.2]	93.4 [83.7–104.7]	0.33
HbA1c, %	6.2 ± 1.7	5.8 ± 1.6	6.1 ± 1.5	6.7 ± 1.9	< 0.001
FBG, mg/dl	6.6 ± 2.7	5.4 ± 1.1	6.0 ± 1.8	8.5 ± 3.6	< 0.001
Cardiovascular medications, n (%)					
Aspirin	1127 (86.6)	342 (78.6)	398 (91.9)	387 (89.2)	< 0.001
P2Y12 inhibitors	1113 (85.5)	338 (77.7)	395 (91.2)	380 (87.6)	< 0.001
Statins	1082 (83.1)	320 (73.6)	385 (88.9)	377 (86.9)	< 0.001
ACEI/ARB	738 (56.7)	217 (49.9)	264 (61.0)	257 (59.2)	0.002
β-blocker	970 (74.5)	292 (67.1)	349 (80.6)	329 (75.8)	< 0.001
Angiographic findings, n (%)					
LM lesion	58 (4.5)	22 (5.1)	22 (5.1)	14 (3.2)	0.31
LAD lesion	970 (74.5)	317 (72.9)	330 (76.2)	323 (74.4)	0.52
LCX lesion	632 (48.5)	189 (43.4)	228 (52.7)	215 (49.5)	0.02
RAD lesion	698 (53.6)	211 (48.5)	229 (52.9)	258 (59.4)	0.005
Multivessel lesion	754 (57.9)	229 (52.6)	259 (59.8)	266 (61.3)	0.02
Diagnosis, n (%)					0.07
AMI	630 (48.4)	193 (44.4)	214 (49.4)	223 (51.4)	
Stable angina	461 (35.4)	173 (39.8)	139 (32.1)	149 (34.3)	
Unstable angina	211 (16.2)	69 (15.9)	80 (18.5)	62 (14.3)	
Gensini score	49.0 [32.0–82.0]	47.0 [31.8–80.0]	49.0 [32.0–82.0]	52.5 [32.0–82.0]	0.32
LVEF, %	58.2 ± 9.3	58.3 ± 9.4	58.4 ± 9.1	58.0 ± 9.4	0.80
Types of PCI, n (%)					0.23
DES	1211 (93.0)	405 (93.1)	409 (94.5)	397 (91.5)	
DEB/PTCA	91 (7.0)	30 (6.9)	24 (5.5)	37 (8.5)	
Follow-up time, months	33 (31–34)	33 (31–34)	32 (31–34)	33 (31–35)	0.39
Target lesion failure, n (%)	43 (3.3)	10 (2.3)	16 (3.7)	17 (3.9)	< 0.001

Data were given as mean ± standard deviation, median with interquartile range or number (percentage)

*EOCAD* early-onset coronary artery disease, *PCI* percutaneous coronary intervention, *TyG* index triglyceride-glucose index;, BMI body mass index, *SBP* systolic blood pressure, *DBP* diastolic blood pressure, *DM* Diabetes Mellitus, *MI* myocardial infarction, *TC* total cholesterol, *TG* triglyceride, *HDL-C* high-density lipoprotein-cholesterol, *LDL-C* low-density lipoprotein-cholesterol, *UA* uric acid, *Scre* serum creatinine, *eGFR* estimated glomerular filtration rate, FBG fasting blood glucose, *HbA1c* Glycosylated Hemoglobin Type A1C, *ACEI/ARB* angiotensin converting enzyme inhibitors/angiotonin receptor blocker, *LM* left main artery, *LAD* left anterior descending artery, *LCX* = left circumflex artery, *RAD* right anterior descending artery, *LVEF* left ventricular ejection fraction, *DES* drug-eluting stent, *DEB* drug-eluting balloon, *PTCA* percutaneous transluminal coronary angioplasty

**Fig. 2:**
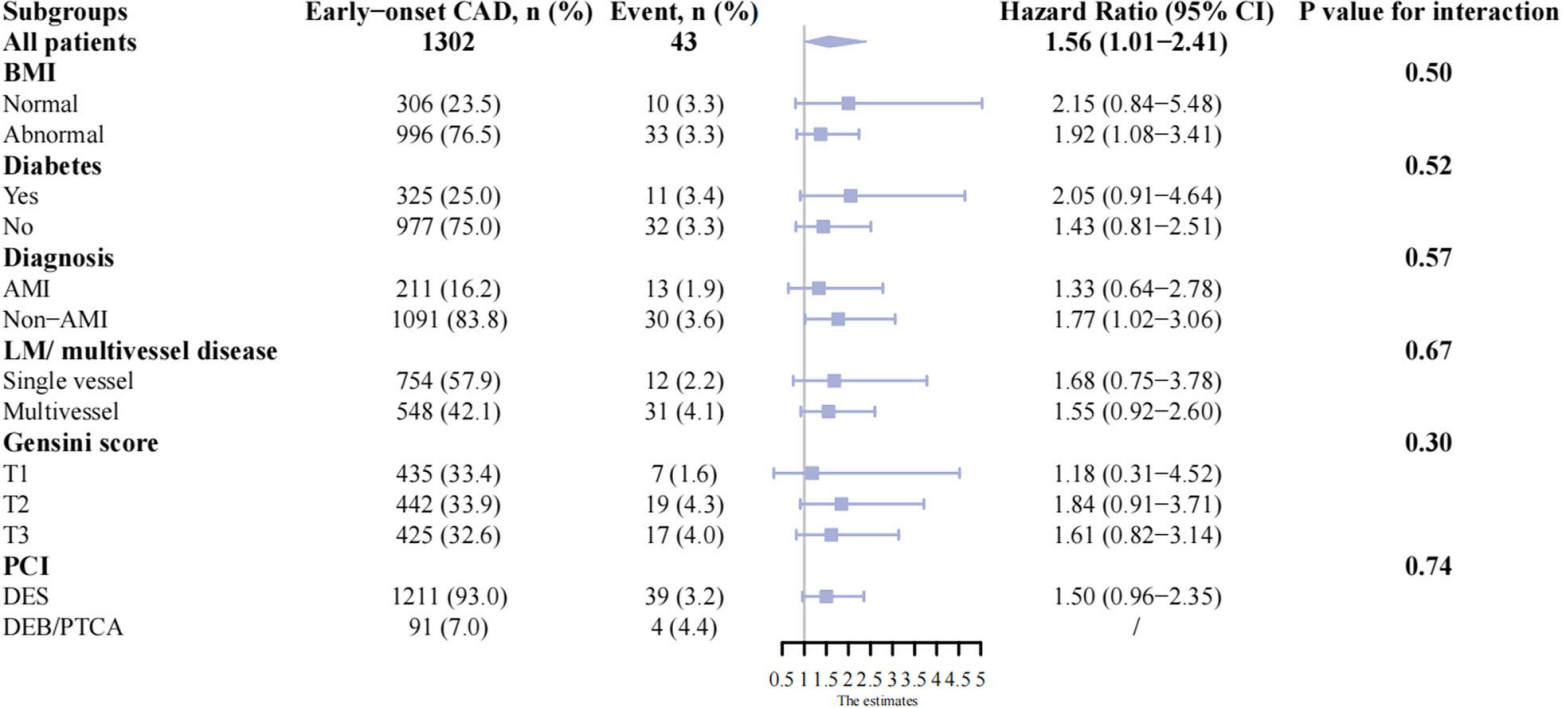
Stratification analysis of the association between TyG index and TLF in different subgroups of patients with EOCAD. *TyG* triglyceride-glucose, *TLF* target lesion failure, *EOCAD* early onset coronary artery disease, BMI body mass index, *AMI* acute myocardial infarction, *LM* left main artery, *PCI* percutaneous coronary intervention, *DES* drug-eluting stent, *DEB* drug-eluting balloon, *PTCA* percutaneous transluminal coronary angioplasty, *HR* hazard ratio, *CI* confidential intervals

During a median of 33 month (IQR: 31–34 months) follow-up, 43 (3.3%) cases had the events of TLF after successful PCI. The median follow-up time across tertile groups of TyG index was comparable (Table 3). The incidence of TLF was increased across tertiles of TyG index (*p* for trend < 0.001, Table 3). Univariate Cox proportional analysis was presented in Additional file 1: Table S5. Only Gensini score and diagnoses of non-AMI on admission had univariate associations with the outcome (*p* < 0.05). In the fully adjusted model, the risk of TLF was increased by 30% (adjusted HR: 1.30; 95% CI 1.01–1.73, *p* = 0.04), per SD increase in the TyG index. Using subjects in the lowest tertile group as control, the fully adjusted HR for TLF was 2.41 (95% CI 0.93–4.60, *p* = 0.08) in the middle and 2.41 (95% CI 1.07–5.42, *p* = 0.03) in the highest tertile group of TyG index (Table 4). The association between the TyG index and TLF was consistent across participants with different categories of BMI, statuses of DM, diagnoses on admission, numbers of diseased vessels, tertiles of Gensini score, and PCI strategies in multivariate analysis (all *p* for interaction ≥ *0.05*) (Fig. 2).

**Table 4:** The association between TyG index and incident TLF among patients with EOCAD

TyG index	Events, n (%)	HR (95% CI)					
		Model1	*p*-value	Model2	*p*-value	Model3	*p*-value
Per SD increase	43 (3.3)	1.16 (0.87–1.55)	0.31	1.30 (0.97–1.74)	0.08	1.30 (1.01–1.73)	0.04
Tri-sectional TyG groups							
Tertile 1	10 (2.3)	1 (Reference)		1 (Reference)		1 (Reference)	
Tertile 2	16 (3.7)	1.62 (0.73–3.56)	0.24	2.04 (0.91–4.57)	0.09	2.04 (0.93–4.60)	0.08
Tertile 3	17 (3.9)	1.72 (0.79–3.750	0.18	2.35 (1.04–5.29)	0.04	2.41 (1.07–5.42)	0.03
*p* for trend		0.18		0.02		0.001	

Model1: unadjusted for covariates

Model2: adjusted for age, sex, body mass index, current smoking, drinking

Model3: adjusted for age, sex, body mass index, current smoking, drinking, hypertension, diabetes mellitus, chronic kidney disease, previous myocardial infarction, Gensini score, inpatient diagnosis, and types of percutaneous coronary intervention

*TyG* triglyceride-glucose, *TLF* target lesion failure, *HR* hazard ratio, *CI* confidential intervals

### Prognostic ability of the TyG index for TLF

**Fig. 3:**
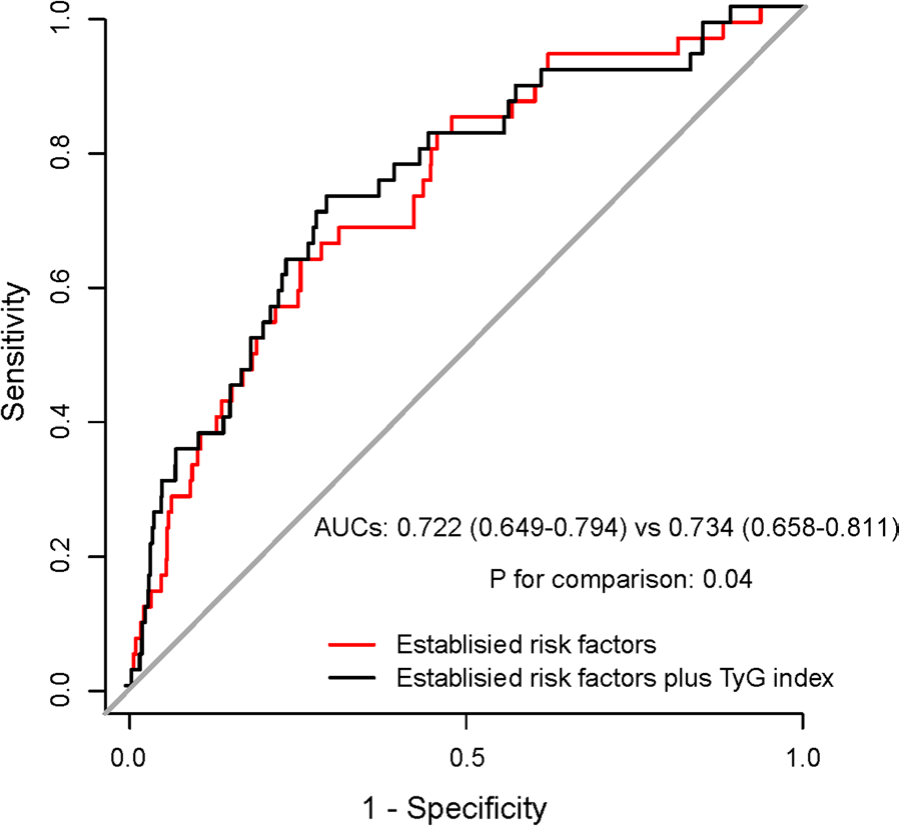
ROC analysis of the predictive value of TyG index for TLF among patients with EOCAD. *ROC* receiver operating characteristic curve, *TyG* index triglyceride-glucose index, *TLF* target lesion failure, *EOCAD* early-onset coronary artery disease, *AUC* area under the curve

The ROC curve for the outcome of TLF showed an AUC of 0.722 (0.649–0.794) when the baseline risk variables, including age, sex, BMI, current smoking, drinking, hypertension, DM, CKD, previous MI, Gensini score, diagnosis, and types of PCI were used alone. The addition of the TyG index to the clinical model provided a considerable increment of AUC to 0.734 (0.658–0.811; likelihood ratio test. *p* = 0.04) (Fig. 3). In addition, the continuous NRI for TLF was 0.132(− 0.065–0.256,* p* = 0.19) and the IDI was 0.004 (− 0.002–0.027,* p* = 0.16), indicating improvement in TLF risk discrimination, although both did not reach the statistical significance.

## Discussion

In this hospital-based clinical study of young patients undergoing coronary angiography, we identified a strong positive association of the TyG index with the presence of severe atherosclerotic obstructive CAD at baseline, and also with the incidence of TLF during a medium period of 33 months (IQR: 31–34 months) after successful PCI. TyG index was significantly correlated with established cardiometabolic risk factors. The addition of the TyG index into known clinical parameters provided improvements in the risk discrimination for EOCAD susceptibility and future TLF. To our knowledge, this is the first multicenter study to inform the role of the TyG index when considering how best to stratify the risk of CAD among young patients.

The risk of CAD was underestimated among young adults < 40 years, and they were merely eligible for preventive treatments according to the current guidelines for the primary prevention of ASCVD [21]. Past studies have validated that the prevalence of DM, hypertension, and hypercholesteremia was lower among young patients with CAD than among older patients [3, 4]. Meanwhile, obesity and metabolic syndrome have been postulated as independent risk factors of prevalent CAD and outcomes in young individuals beyond traditional risk factors [6]. IR is a state of reduced sensitivity and impaired response to insulin action, which has been identified as a pathogenic driver of DM and metabolic syndrome [8]. On the other hand, it was well known that IR itself plays a pathogenic role in the formation and aggravation of atherosclerotic plaque independent of traditional risk factors [10]. TyG index is a low-cost and readily available tool to identify IR among patients with and without DM, and has been proven to outperform the homeostasis model assessment estimated insulin resistance test (HOMA-IR) index, using hyperinsulinemic-euglycemic clamp test as gold standard [11, 22, 23].

The independent association between the TyG index with CAD has been described in both middle-aged and elderly patients [12–14]. However, few studies have examined the association between TyG index and CAD disease susceptibility among young adults. In a small cross-sectional study, each 1-unit increase in TyG index was associated with a 2.06-fold increased risk of angiographically proven CAD among 424 patients with non-alcoholic fatty liver disease [24]. The results from 15 cohort studies were reported in two separate meta-analyses that identified a strong association between elevated TyG index and incident ASCVD in the general population [13, 14]. This association appeared independent of conventional risk factors, despite the heterogeneity in those study results confines interpretations in this regard. Furthermore, the TyG index is an independent predictor of subclinical CAD in populations without established coronary risk factors [25]. In the present study, we demonstrated that a higher level of TyG index increased the likelihood of severe atherosclerotic obstructive CAD independent of conventional risk factors among patients aged between 18 and 45 years. When AUCs were examined for the diagnostic ability for EOCAD, the diagnostic power of the TyG index is moderate, and introducing the TyG index into the preexisting clinical model would confer incremental benefit in risk classification among young adults.

The predictive value of the TyG-index is most likely related to their ability to signal early cardiometabolic disturbance among apparently healthy young adults. One recent study in China that examined the prognostic significance of the TyG index for major adverse cardiovaslucar events in young patients (male < 45 years, female < 55 years) with angiographically proven CAD has revealed the significant correlations of the TyG index with traditional cardiovascular risk factors, although this study was limited by its single-center and retrospective study design, as well as the small sample size (n = 526) [26]. Similarly, our results from larger samples of young subjects at 38 hospitals in China also confirmed that higher levels of TyG index were closely correlated to higher burden traditional risk factors among EOCAD patients, including obesity and obesity-related metabolic abnormalities, such as increased levels of Hb1Ac, non-HDL-c, TC, SBP and DBP, and decreased levels of HDL-c. Since IR may precede these cardiometabolic disorders for years, our shreds of evidence support the notion that screening for higher TyG-index has great potential for early identification of the CAD risk among young individuals.

As young patients in our cohort were selected for severe atherosclerotic obstructive CAD (> 70% luminal stenosis in main arteries), they represent a relatively homogenous high-risk population. TyG-index may provide greater discrimination of atherosclerotic disease severity in a more heterogeneous population such as in previous studies that included young and elderly patients with CAD (> 50% luminal stenosis in main arteries) [24, 26, 27]. Consequently, no significant correlation was observed between the TyG index and the Genisni score in the present investigation.

Early-onset CAD is not a benign condition [4–7]. Most patients (86.1%) in our cohort were treated with PCI at a young age. Developing TLF in these patients would be catastrophic, considering the longer life expectancy at risk for recurrent ischemic events [6, 7]. A retrospective cohort study that enrolled 1574 patients with acute coronary syndrome who underwent successful stent implantation revealed an improved predictive power of the TyG index for in-stent restenosis beyond a model of established risk factors. In the present study,we prospectively demonstrated that the TyG index either as a continuous or categorical variable, was independently associated with the increased risk of incident TLF after adjusting for confounding factors. The association was not substantially different in various subgroups of BMI, status of DM or acute MI, coronary disease severity, and PCI strategies in the subgroup analysis. Our data demonstrated a considerable improvement in the prognostic power for incident TLF when adding the TyG index to conventional clinical parameters, and the reclassification ability was improved by 13.2%, although the NRI was not statistically significant due to the lower event rate.

Sex disparities in the TyG index remain uncertain. Some studies found a link between the TyG index and ASCVD risk in females, but not in males [28, 29]. However, in the sensitivity analysis, we confirmed that restricting subjects to males did not substantively alter the association between the TyG index and the likelihood of EOCAD.

## Limitations

This study does have important limitations. To begin, the causal association of the TyG index and disease susceptibility and outcome is difficult to establish due to the observational nature of the study design. Moreover, we eliminated cases with extremely high TG or FBG to account for the factors like stress hyperglycemia and familial hypertriglyceridemia. However, residual confounders including medications, physical activities, dietary habits and family history of CAD may exist. What’s more, the baseline case–control study was conducted using 1:1 matching only by age, while sex was unable to be further matched. Furthermore, all studies using the TyG index suffer from limitations related to biological variability and intraindividual variation. Nonetheless, the cumulative effect of the TyG index over time seemed to be better than the TyG index at baseline in predicting CAD risk [12]. Unfortunately, we lack the follow-up data on the TyG index after discharge. Besides, given that fasting insulin was not measured in most participants, the present study was not powered to compare the predictive ability of the TyG index with that of other IR metrics, such as HOMA-IR. However, the TyG index has been reported superior to HOMA-IR index in predicting arteriosclerosis [30, 31] and CVD outcomes [32]. In addition, we had few patients with TLF and a relatively short period of follow-up. Therefore, the predictive ability of the TyG index for the risk of TLF and other clinically relevant outcomes, such as cardiovascular death or major adverse cardiovascular events, could not be assessed reliably in our study. More importantly, data on intracoronary imaging (intravascular ultrasound or optical coherence tomography), follow-up medications and status of modifiable risk factors controlling (smoking cessation, weight controls, BP, Hb1Ac, and lipid profiles) were not available, limiting our ability to determine whether the association of TyG index with TLF remains independent of these factors. Finally, in this hospital-based study, our target population was young, hospitalized patients referred to angiographic coronary evaluation and predominately male, and therefore we need extra caution regarding the generalizability of these findings to the general young populations.

## Conclusion

This is the first multicenter study to date to assess the association between the TyG index and the risk of prevalent EOCAD and incident TLF. In the current study, the TyG index, a surrogate of IR, presented an incremental diagnostic power for EOCAD beyond well-known risk factors. The prognostic value of the TyG index is moderate for TLF, and incorporating the TyG index would improve the predictive accuracy of the conventional risk model. Our results suggested the usefulness of the TyG index in screening subjects who have severe atherosclerotic CAD among young adults and in identifying EOCAD patients who are at high risk of future TLF, so that intensive strategies can be provided.

### Supplementary Information

**Additional file 1: Table S1.** Baseline characteristics of male participants with and without EOCAD. **Table S2.** Baseline characteristics between controls and cases from different locations. **Table S3.** Correlations between TyG index and traditional cardiovascular risk factors. **Table S4.** The association between TyG index and the prevalent EOCAD in males. **Table S5.** Univariate Cox regression analysis for TLF in EOCAD. **Figure S1.** ROC analysis of the diagnostic ability of TyG index at hospitalization to identify EOCAD in males.

## Data Availability

The original data analyzed during this study are included in the article and its Additional file. Further inquiries can be directed to the corresponding author on reasonable request.

## Abbreviations

ASCVD: Atherosclerotic cardiovascular disease
AUC: Area under the curve
BMI: Body mass index
CI: Confidence interval
CKD: Chronic kidney disease
DBP: Diastolic blood pressure
DM: Diabetes Mellitus
eGFR: Estimated glomerular filtration rates
EOCAD: Early-onset coronary artery disease
FBG: Fasting blood glucose
HbA1c: Hemoglobin A1c
HDL-c: High-density lipoprotein cholesterol
HOMA: Homeostasis model assessment estimated insulin resistance
IDI: Integrated discrimination improvement
IR: Insulin resistance
LM: Left main
LVEF: Left ventricular ejection fraction
MI: Myocardial infarction
NRI: Net reclassification improvement
OR: Odds ratios
PCI: Percutaneous coronary intervention
ROC: Receiver-operating characteristic curve
SBP: Systolic blood pressure
SD: Standard deviation
TC: Total cholesterol
TG: Triglyceride
TyG: Triglyceride-glucose
TLF: Target lesion failure
