# Supplementary material for: Triglyceride-glucose index is associated with severe obstructive coronary artery disease and atherosclerotic target lesion failure among young adults
Source: Cardiovasc Diabetol. 2023 Oct 21;22:283. doi: 10.1186/s12933-023-02004-1 (PMC10590519; doi:10.1186/s12933-023-02004-1)
Supplement: Supplementary file 1 — Additional file 1: Table S1. Baseline characteristics of male participants with and without EOCAD. Table S2. Baseline characteristics between controls and cases from different locations. Table S3. Correlations between TyG index and traditional cardiovascular risk factors. Table S4. The association between TyG index and the prevalent EOCAD in males. Table S5. Univariate Cox regression analysis for TLF in EOCAD. Figure S1. ROC analysis of the diagnostic ability of TyG index at hospitalization to identify EOCAD in males. [file 12933_2023_2004_MOESM1_ESM.docx]

**Additional file**

| **Table S1. Baseline characteristics of male participants with and without EOCAD.** | | | | |
| --- | --- | --- | --- | --- |
|  | **Overall**  **N=1684** | **EOCAD**  **N=842** | **Control**  **N=842** | ***p*-value** |
| **General conditions** |  |  |  |  |
| Age, year | 38.0±4.9 | 38.0±4.9 | 38.0±4.9 | 0.91 |
| BMI, kg/m^2^ | 26.1±4.1 | 27.4±4.1 | 24.8±3.6 | <0.001 |
| Body shape, n (%) |  |  |  | <0.001 |
| Normal | 562 (34.1) | 203 (24.5) | 359 (43.9) |  |
| Overweight | 507 (30.8) | 202 (24.3) | 305 (37.3) |  |
| Obesity | 578 (35.1) | 425 (51.2) | 153 (18.7) |  |
| Heart rate | 78.5±13.8 | 78.3±13.0 | 78.8±14.6 | 0.48 |
| SBP, mmHg | 127.2±17.3 | 128.8±19.7 | 125.6±14.3 | <0.001 |
| DBP, mmHg | 80.9±12.7 | 81.5±14.3 | 80.3±10.9 | 0.052 |
| Smoking n (%) |  |  |  | <0.001 |
| Never | 749 (44.5) | 181 (21.5) | 568 (67.5) |  |
| Former | 282 (16.7) | 169 (20.1) | 113 (13.4) |  |
| Current | 653 (38.8) | 492 (58.4) | 161 (19.1) |  |
| Drinking n (%) | 486 (28.9) | 395 (46.9) | 91 (10.8) | <0.001 |
| **Comorbidities** n (%) |  |  |  |  |
| Hypertension | 647 (38.4) | 385 (45.7) | 262 (31.1) | <0.001 |
| DM | 306 (18.2) | 216 (25.7) | 90 (10.7) | <0.001 |
| Dyslipidemia | 983 (58.4) | 514 (61.0) | 469 (55.7) | 0.03 |
| Chronic kidney disease | 37 (2.2) | 23 (2.7) | 14 (1.7) | 0.18 |
| History of myocardial infarction | 154 (9.1) | 154 (18.3) | 0 (0.0) | <0.001 |
| **Laboratory results** |  |  |  |  |
| TC, mmol/l | 4.4±1.4 | 4.5±1.8 | 4.3±0.9 | 0.03 |
| TG, mmol/l | 2.3±1.6 | 2.5±1.8 | 2.1±1.5 | <0.001 |
| HDL-C, mmol/l | 1.0±0.3 | 1.0±0.3 | 1.1±0.3 | <0.001 |
| LDL-C, mmol/l | 2.5±1.3 | 2.7±1.7 | 2.4±0.8 | <0.001 |
| non-HDL-C, mmol/l | 3.4±1.4 | 3.5±1.7 | 3.2±0.9 | <0.001 |
| UA, mg/dl | 6.5±1.6 | 6.5±1.7 | 6.4±1.5 | 0.04 |
| Scre, mg/dl | 0.9±0.3 | 0.9±0.2 | 1.0±0.4 | 0.001 |
| eGFR, mL/min/1.73 m^2^ | 90.5 [81.6-100.2] | 92.7 [82.8-104.0] | 88.8 [80.8-96.7] | <0.001 |
| FBG, mg/dl | 6.0±2.2 | 6.6±2.7 | 5.3±1.3 | <0.001 |
| HbA1c, % | 5.8±1.4 | 6.2±1.7 | 5.4±0.7 | <0.001 |
| TyG index | 9.1±0.7 | 9.2±0.7 | 8.9±0.7 | <0.001 |
| Data were given as mean ± standard deviation, median with interquartile range or number (percentage). EOCAD=early-onset coronary artery disease; BMI=body mass index; SBP=systolic blood pressure; DBP=diastolic blood pressure; DM=Diabetes Mellitus; TC=total cholesterol; TG=triglyceride; HDL-C=high-density lipoprotein-cholesterol; LDL-C=low-density lipoprotein-cholesterol; UA=uric acid; Scre=serum creatinine; eGFR=estimated glomerular filtration rate; FBG=fasting blood glucose; HbA1c=Glycosylated Hemoglobin Type A1C; TyG =triglyceride-glucose. | | | | |

Table S2. Baseline characteristics between controls and cases from different locations.

|  | Cases from Zhongshan-Hospital  N=732 | Cases Not from Zhongshan-Hospital  N=781 | Controls  N=1513 |
| --- | --- | --- | --- |
| General conditions |  |  |  |
| Age, year ^*^ | 39.9±4.31 | 39.3±4.54 | 39.0±4.40 |
| Female, n (%) ^*#^ | 34 (4.64) | 35 (4.48) | 811 (53.6) |
| BMI, kg/m2 ^*#^ | 26.5±3.52 | 26.4±3.57 | 23.6±3.74 |
| Body weight group, n (%) ^*#^ |  |  |  |
| Normal | 149 (21.1) | 193 (25.0) | 878 (59.6) |
| Overweight | 334 (47.4) | 351 (45.4) | 386 (26.2) |
| Obesity | 222 (31.5) | 229 (29.6) | 210 (14.2) |
| Heart rate, beat per minute ^*^ | 76.0±11.7 | 79.8±14.2 | 78.9±13.5 |
| SBP, mmHg ^*#^ | 125±16.7 | 131±20.7 | 123±14.7 |
| DBP, mmHg ^#^ | 79.2±11.7 | 83.1±15.5 | 78.1±10.8 |
| Smoking, n (%) ^*#^ |  |  |  |
| Never | 219 (29.9) | 163 (20.9) | 1288 (85.1) |
| Former | 204 (27.9) | 104 (13.3) | 96 (6.35) |
| Current | 309 (42.2) | 514 (65.8) | 129 (8.53) |
| Drinking, n (%) ^*#^ | 275 (37.6) | 387 (49.6) | 76 (5.02) |
| Comorbidities, n (%) ^*#^ |  |  |  |
| Hypertension ^*#^ | 310 (42.3) | 359 (46.0) | 377 (24.9) |
| DM ^*#^ | 198 (27.0) | 178 (22.8) | 151 (9.98) |
| Dyslipidemia ^*#^ | 456 (62.3) | 417 (53.4) | 592 (39.1) |
| Chronic kidney disease ^*#^ | 25 (3.42) | 14 (1.79) | 21 (1.39) |
| Laboratory results |  |  |  |
| TC, mmol/l ^*#^ | 3.99±1.74 | 4.69±1.43 | 4.20±0.84 |
| TG, mmol/l ^*#^ | 2.30±1.63 | 2.37±1.70 | 1.70±1.31 |
| HDL-C, mmol/l ^*#^ | 1.04±0.61 | 0.99±0.34 | 1.23±0.37 |
| LDL-C, mmol/l ^#^ | 2.37±1.81 | 2.92±1.18 | 2.25±0.71 |
| non-HDL-C, mmol/l ^*#^ | 3.12±1.64 | 3.70±1.41 | 2.97±0.85 |
| UA, mg/dl ^*#^ | 6.41±1.57 | 6.62±1.77 | 5.41±1.69 |
| Scre, mg/dl ^*#^ | 0.96±0.43 | 0.84±0.23 | 0.81±0.31 |
| eGFR, mL/min/1.73 m2 ^*#^ | 88.2 [79.4;96.9] | 97.4 [86.5;109] | 90.4 [82.6;97.9] |
| HbA1c, % ^*#^ | 6.06±1.68 | 6.38±1.76 | 5.39±0.73 |
| FBG, mg/dl ^*#^ | 6.60±2.82 | 6.66±2.71 | 5.33±1.22 |
| TyG ^*#^ | 9.13±0.78 | 9.18±0.74 | 8.66±0.68 |
| Data were given as mean ± standard deviation, median with interquartile range or number (percentage).  The ^*^ represents a comparison between cases from Zhongshan Hospital and controls with p < 0.05.  The ^#^ represents a comparison between cases Not from Zhongshan Hospital and controls with p < 0.05.  EOCAD=early-onset coronary artery disease; BMI=body mass index; SBP=systolic blood pressure; DBP=diastolic blood pressure; DM=diabetes mellitus; MI=myocardial infarction; TC=total cholesterol; TG=triglyceride; HDL-C=high-density lipoprotein-cholesterol; LDL-C=low-density lipoprotein-cholesterol; UA=uric acid; Scre=serum creatinine; eGFR=estimated glomerular filtration rate; HbA1c=Glycosylated Hemoglobin Type A1c; FBG=fasting blood glucose; TyG index=triglyceride-glucose index. | | | |

| **Table S3. Correlations between TyG index and traditional cardiovascular risk factors** | | |
| --- | --- | --- |
| **Variable** | **Correlation coefficient** | ***p*-value** |
| Age | -0.015 | 0.29 |
| BMI | 0.190 | <0.001 |
| SBP | 0.124 | <0.001 |
| DBP | 0.116 | <0.001 |
| TC | 0.236 | <0.001 |
| HDL-C | -0.238 | <0.001 |
| LDL-C | -0.032 | 0.25 |
| non-HDL-C | 0.287 | <0.001 |
| HbA1c | 0.293 | <0.001 |
| UA | 0.023 | 0.40 |
| Scre | 0.010 | 0.73 |
| eGFR | 0.035 | 0.21 |
| Gensini score | 0.040 | 0.15 |
| LVEF | -0.045 | 0.11 |
| Correlation coefficient presented with the coefficient of Spearman or Pearson test  TyG=triglyceride-glucose;BMI=body mass index; SBP=systolic blood pressure; DBP=diastolic blood pressure; TC=total cholesterol; HDL-C=high-density lipoprotein-cholesterol; LDL-C=low-density lipoprotein-cholesterol; HbA1c Glycosylated Hemoglobin Type A1C; UA=uric acid; Scre=serum creatinine; eGFR=estimated glomerular filtration rate; LVEF=left ventricular ejection fraction. | | |

| **Table S4. The association between TyG index and the prevalent EOCAD in males.** | | | | | | |
| --- | --- | --- | --- | --- | --- | --- |
| **TyG index** | **OR (95%CI)** | | | | | |
|  | **Model1** | ***p*-value** | **Model2** | ***p*-value** | **Model3** | ***p*-value** |
| Per 1 SD increase | 1.59 (1.43-1.76) | <0.001 | 1.24 (1.10-1.41) | <0.001 | 1.44 (1.25-1.67) | <0.001 |
| Tri-sectional TyG groups |  |  |  |  |  |  |
| Tertile 1 | 1 (Reference) |  | 1 (Reference) |  | 1 (Reference) |  |
| Tertile 2 | 1.35 (1.06-1.71) | 0.01 | 1.15 (0.88-1.51) | 0.32 | 1.22 (0.90-1.67) | 0.19 |
| Tertile 3 | 2.57 (2.02-3.28) | <0.001 | 2.12 (1.61-2.79) | <0.001 | 2.06 (1.45-2.93) | <0.001 |
| *p* for trend | 0.001 |  | 0.003 |  | 0.01 |  |
| Model1: unadjusted for covariates;  Model2: adjusted for age, body mass index, current smoking, drinking;  Model3: adjusted for age, body mass index, current smoking, drinking, hypertension, diabetes mellitus;  TyG=triglyceride-glucose; EOCAD=early-onset coronary artery disease; OR=odds ratio; CI=confidence intervals. | | | | | | |

| **Table S5. Univariate Cox regression analysis for TLF in EOCAD** | | |
| --- | --- | --- |
|  | **HR (95% CI)** | ***p*-value** |
| Age | 1.05 (0.97-1.13) | 0.22 |
| Female | 2.42 (0.87-6.78) | 0.09 |
| BMI | 0.93 (0.85-1.02) | 0.11 |
| SBP | 0.99 (0.98-1.01) | 0.30 |
| DBP | 0.99 (0.97-1.01) | 0.42 |
| TG | 1.04 (0.88-1.23) | 0.66 |
| HDL-C | 0.84 (0.38-1.85) | 0.66 |
| LDL-C | 0.90 (0.71-1.14) | 0.36 |
| nHDL-C | 1.13 (0.96-1.35) | 0.16 |
| UA | 0.88 (0.73-1.07) | 0.21 |
| HbA1c | 0.97 (0.81-1.16) | 0.72 |
| eGFR | 0.98 (0.97-1.001) | 0.07 |
| Gensini score | 1.008 (1.003-1.015) | 0.01 |
| LVEF | 1.00 (0.97-1.03) | 0.98 |
| Hypertension | 0.85 (0.46-1.57) | 0.61 |
| DM | 1.03 (0.52-2.04) | 0.93 |
| Dyslipidemia | 1.33 (0.71-2.48) | 0.38 |
| Chronic kidney disease | 0.96 (0.13-6.98) | 0.97 |
| History of previous MI | 1.71 (0.88-3.33) | 0.12 |
| Drinking | 0.86 (0.46-1.58) | 0.61 |
| LM lesion | 1.62 (0.50-5.25) | 0.42 |
| LAD lesion | 1.29 (0.62-2.70) | 0.49 |
| LCX lesion | 1.49 (0.81-2.73) | 0.20 |
| RAD lesion | 1.47 (0.79-2.74) | 0.22 |
| **Diagnosis** |  |  |
| AMI | 1.00 |  |
| Non-AMI | 2.20 (1.15-4.21) | 0.02 |
| **PCI** |  |  |
| DES | 1.00 |  |
| DEB/PTCA | 0.73 (0.26-2.04) | 0.55 |
| **Cardiovascular medications** |  |  |
| Aspirin | 2.08 (0.64-6.72) | 0.22 |
| P2Y12 inhibitors | 7.23 (0.99-52.51) | 0.05 |
| Statins | 2.75 (0.85-8.88) | 0.09 |
| ACEI/ARB | 1.78 (0.93-3.41) | 0.08 |
| β-blocker | 2.63 (1.00-6.69) | 0.05 |
| EOCAD=early-onset coronary artery disease; TLF=target lesion failure; HR=hazard ratio; CI=confidential intervals; BMI=body mass index; SBP=systolic blood pressure; DBP=diastolic blood pressure; TG=triglyceride; HDL-C=high-density lipoprotein-cholesterol; LDL-C=low-density lipoprotein-cholesterol; UA=uric acid; HbA1c=Glycosylated Hemoglobin Type A1C; eGFR=estimated glomerular filtration rate; LVEF=left ventricular ejection fraction; DM=diabetes mellitus; MI=myocardial infarction; LM=left main artery; LAD=left anterior descending artery; LCX=left circumflex artery; RAD=right anterior descending artery; PCI=percutaneous coronary intervention; DES=drug-eluting stent, DEB=drug-eluting balloon, PTCA=percutaneous transluminal coronary angioplasty; ACEI/ARB=angiotensin converting enzyme inhibitors/angiotonin receptor blocker. | | |

**Figure S1. ROC analysis of the diagnostic ability of TyG index at hospitalization to identify EOCAD in males**


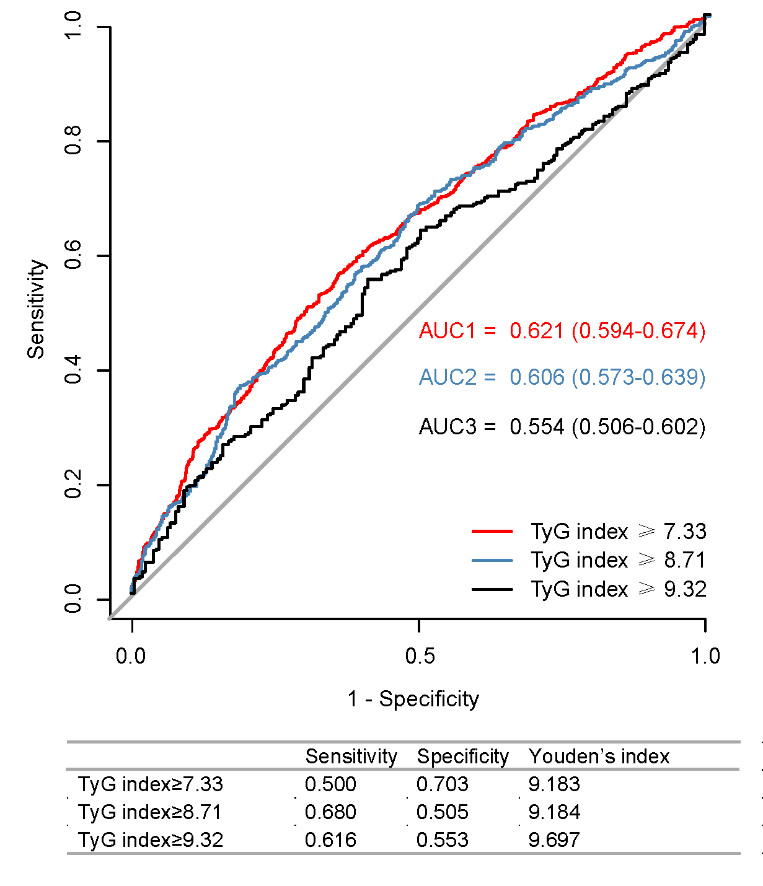


ROC=receiver operating characteristic curve; TyG=triglyceride-glucose index; EOCAD=early-onset coronary artery disease; AUC=area under curve.
